# Quantifying dosage of physical therapy using lower body kinematics: a longitudinal pilot study on early post-stroke individuals

**DOI:** 10.1186/s12984-020-0655-0

**Published:** 2020-02-07

**Authors:** Sung Yul Shin, Robert K. Lee, Patrick Spicer, James Sulzer

**Affiliations:** 1 Department of Mechanical Engineering, University of Texas at Austin, 204 E Dean Keeton St, Austin, TX 78712 USA; 2 St. David’s Rehabilitation Hospital, St. David’s Medical Center, 919 E 32nd St, Austin, TX 78705 USA; 3 Seton Brain and Spine Institute, Ascension Texas, 1201 W 38th St, Austin, TX 78705 USA

**Keywords:** Gait, Stroke, Therapy, Dosage, Inertial measurement units

## Abstract

**Background:**

While therapy is an important part of the recovery process, there is a lack of quantitative data detailing the “dosage” of therapy received due to the limitations on in/outpatient accessibility and mobility. Advances in wearable sensor technology have allowed us to obtain an unprecedented glimpse into joint-level kinematics in an unobtrusive manner. The objective of this observational longitudinal pilot study was to evaluate the relations between lower body joint kinematics during therapy and functional gait recovery over the first three months after stroke.

**Methods:**

Six individuals with subacute stroke (< 1 month) were monitored for a total of 59 one-hour physical therapy sessions including gait and non-gait activities. Participants donned a heart rate monitor and an inertial motion capture system to measure full lower body joint kinematics during each therapy session. Linear mixed regression models were used to examine relations between functional gait recovery (speed) and activity features including total joint displacements, defined as amount of motion (AoM), step number, change in heart rate (*∆HR*), and types of tasks performed.

**Results:**

All activity features including AoM, step number, types of tasks performed (all *p* < 0.01), and *∆HR* (*p* < 0.05) showed strong associations with gait speed. However, AoM (*R*^*2*^ = 32.1%) revealed the greatest explained variance followed by step number (*R*^*2*^ = 14.1%), types of tasks performed (*R*^*2*^ = 8.0%) and *∆HR* (*R*^*2*^ = 5.8%). These relations included both gait and non-gait tasks. Contrary to our expectations, we did not observe a greater relation of functional recovery to motion in the impaired limb (*R*^*2*^ = 27.8%) compared to the unimpaired limb (*R*^*2*^ = 32.9%).

**Conclusions:**

This proof-of-concept study shows that recording joint kinematics during gait therapy longitudinally after stroke is feasible and yields important information for the recovery process. These initial results suggest that compared to step number, more holistic outcome measures such as joint motions may be more informative and help elucidate the dosage of therapy.

## Background

The first months following neurological injury such as stroke, known as the subacute stage, are the most critical to sensorimotor recovery of locomotor function [1]. Despite the importance of this initial stage, we lack detailed information of the patient therapy experience, which varies by therapist preference, patients’ abilities, and insurance coverage. Meta-analyses suggest that the initial impairment level is the strongest predictor of final level of functional recovery after three months [2, 3]. These results could imply that spontaneous mechanisms dictate recovery, or on the other hand, could imply that our measures of recovery are too coarse to be useful predictors.

Unlike pharmacological interventions, the dosage of physical therapy remains unmeasured or unspecified making assessment of its utility exceedingly difficult [4, 5]. The advent of wearable sensor technology, specifically using accelerometers, has vastly improved our ability to monitor motions in an in/outpatient environment [6–9]. In lower limb recovery, the accelerometers were placed on the ankle to track the number of steps during and beyond training sessions [7, 8]. The researchers observed that the number of steps significantly correlated with gait outcome measures [7] more than the intensity (heart rate reserve) [8]. While the number of steps could be an accurate measure of therapy dosage, more detailed information about individual joint motions remain unknown, although are possibly critical to understanding dosage.

Measuring joint kinematics in a clinical environment is challenging due to the limitations on in/outpatient accessibility and mobility. Typical motion capture technology requires a light-controlled, fixed environment and a lengthy setup time. The recent introduction of wireless motion capture based on inertial measurement units (IMUs) provides a flexible, more user-friendly alternative with comparable accuracy to optical methods [10, 11]. IMUs are portable, low-profile devices composed of accelerometers, gyroscopes and magnetometer measuring linear acceleration, angular velocity and angular orientation relative to earth’s magnetic field, respectively. IMUs provide three-dimensional angular motion of individual body segments as well as joint angle trajectories. Importantly, unlike optical motion capture systems, IMUs are minimally obtrusive to therapy with a short setup time and ability to monitor motion indoors and even outdoors, making them feasible for inpatient and outpatient studies.

The objective of this observational pilot study is to examine the relations between joint kinematics during therapy and functional recovery over the early stage of recovery. To achieve this, we longitudinally monitored full lower body kinematics and heart rate of six post-stroke individuals from the first inpatient gait therapy session until the 12th week of therapy in an outpatient setting. Amount of motion (AoM), the total amount of joint displacements measured from inertial motion capture, was used as our primary dosage of therapy feature. Our main functional outcome measure was gait speed recorded during therapy sessions. We expected to find that the AoM would better represent gait recovery than number of steps due to the richer data provided. We also expected to find that training with greater focus on the impaired side would be associated with greater functional improvements [12].

To our knowledge, this proof-of-concept work is the first longitudinal study measuring full lower body kinematics during physical therapy sessions on subacute post-stroke individuals. The information gleaned from this study will provide a more nuanced picture of post-stroke recovery during the early stage, leading towards an improved understanding of the benefits of physical therapy.

## Methods

### Participants

We recruited nine individuals with subacute stroke (< 1 month) to participate in this study approved by University of Texas Institutional Review Board and St. David’s Medical Center, Austin, TX. Among nine patients, six individuals were longitudinally monitored for a total of 59 one-hour physical therapy sessions consisting of gait and non-gait activities. Individuals ranged in age and impairment level (see Table 1). Inclusion criteria of this study were: ischemic cerebral infarction based on MRI data, hemiparesis, premorbidly independent, within two weeks following injury or as soon as able to participate in gait training determined by the physical therapist. Exclusion criteria include cerebellar damage, prior stroke, stroke-related pain syndromes, or functionally relevant neuromuscular impairments.

**Table 1:** Demographics and clinical characteristics of participants

Participant #	P1	P2	P3	P4	P5	P6
Age (years)	61	59	68	68	52	27
Sex	M	M	M	M	F	M
BMI (kg/cm^2^)	29.6	25.8	28.7	28.2	25.8	40.5
Affected side	L	L	R	R	L	R
1st recording after admission (days)	15	5	6	7	20	31
Recordings (sessions)	12	7	11	5	12	12
Ankle foot orthosis (used sessions)	–	6	11	–	–	12
Assistive device (used sessions)	1	7	3	5	10	11
Baseline impairment (admission motor FIM score)	18	26	31	22	22	16

*BMI* body mass index, *FIM* functional independence measure, *M* male, *F* female, *L* left, *R* right

Participants were allowed to use any assistive devices including wheelchair, body weight support, walker, cane or ankle foot orthosis (AFO) during sessions if necessary (see Table 1). Prior to the first session, the experimenter and therapist explained all the experimental procedures to each participant and obtained informed consent. We began monitoring therapy as soon as participants were capable of beginning gait therapy, i.e. as soon as the participants were able to stand with the therapist’s assistance and ready for walking. Three participants did not complete the full dataset; one participant did not complete the full 12 therapy sessions due to discharge to a different hospital (P2), one could only complete 11 sessions due to lack of insurance coverage (P3), and one was discharged to home therapy (P4). Three participants were excluded from the dataset because two of them were not able to walk before discharge and one discharged before the recording session.

### Experimental setup and protocol

Our goal was to consecutively monitor conventional physical therapy sessions with minimal intrusion. We targeted a maximum of 12 recording sessions over the course of the first three months throughout the inpatient to outpatient phases. Our recording session frequency was greater in the earlier stages of recovery, i.e. two/week for three weeks, one/week for four weeks, then one every other week. For each session, we attached seven commercial IMU motion sensors (XSens, Enschede, The Netherlands) on the pelvis and bilaterally on thighs, shanks and feet.

IMUs recorded three-dimensional orientations of all lower body segments as well as joint angle trajectories at a sampling rate of 60 Hz. Participants also donned a heart rate monitor (Polar Electro, NY) on the chest to record heart rate sampled at 1 Hz. Data was transmitted wirelessly to a commercial Latitude E5470 laptop (Dell, TX).

**Fig. 1:**
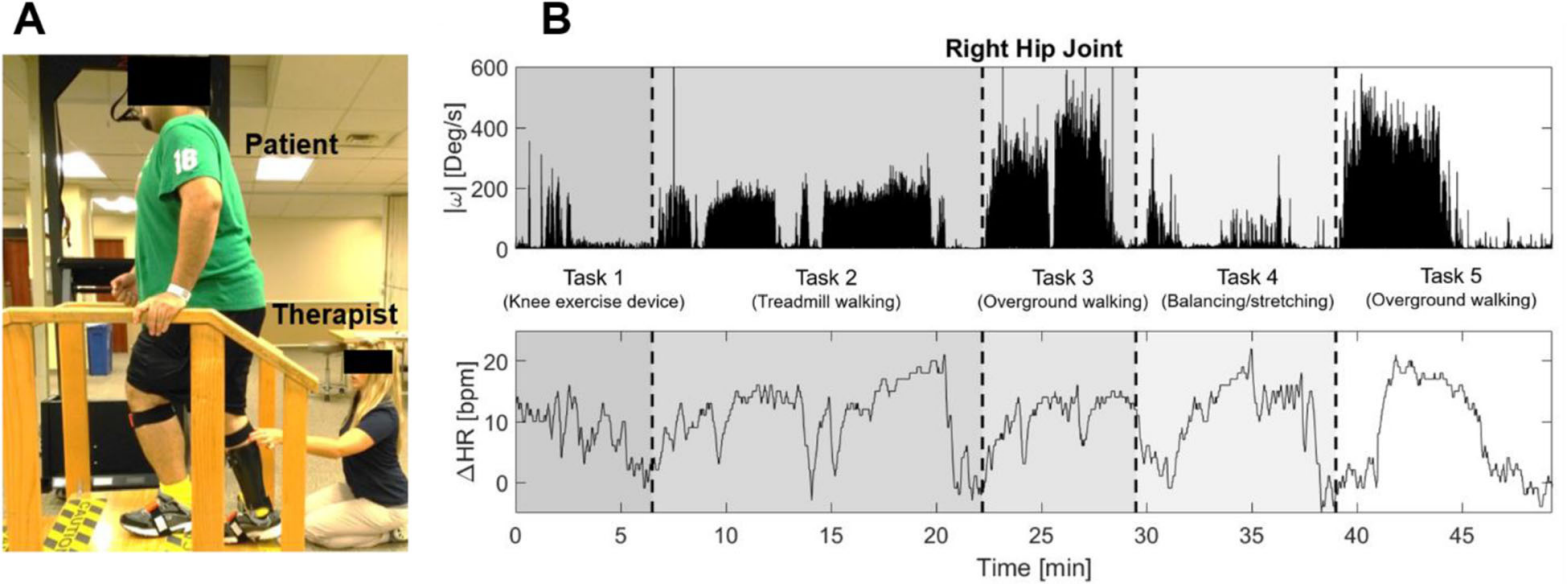
**a**, Capture of a patient receiving conventional physical therapy while wearing IMU and heart rate (underneath t-shirt) sensors. **b**, Absolute angular velocity, ∣ω∣, at right hip joint obtained from IMU sensors (top graph) and heart rate change, *∆HR*, from baseline (bottom graph) during a 1-h physical therapy session

The total setup time took approximately 10 to 20 min. All setup was performed immediately prior to the normally scheduled physical therapy sessions. After donning sensors, participants were asked to maintain a straight standing position for five seconds to calibrate the system as recommended by the manufacturer. Therapist assistance was provided for this calibration as necessary. Following calibration, participants performed a typical 1-h training session under the supervision of a physical therapist (see Fig. 1a). The experimenter used a cart to follow the participant and therapist to maintain wireless communication of sensor signals. The experimenter noted all therapy activities. Altogether, the setup time and donning the sensors provided minimal disruption to the typical therapy regimen. Each therapy session incorporated a number of tasks aside from overground walking and body weight supported treadmill training. Sessions included activities of daily living such as transferring to wheelchair and stair climbing, strengthening activities such as an exercise bike and weight bearing, as well as coordination activities such as stepping and balancing. After the session, all sensors were removed from the participant.

### Feature extraction and outcomes

For each session, all features of therapy dosage and gait outcome measures were extracted from the recorded data. Custom software was written in MATLAB R2016a (Mathworks, Inc., Natick, MA) to calculate the features and outcomes. Figure 1b shows an example of recorded data including absolute angular velocity of right hip joint and heart rate change from baseline over a whole 1-h physical therapy session. Sensor data and notes taken by the experimenter were used to extract the features of therapy dosage and the gait outcomes as described below.

We categorized the features of dosage into amount, intensity and variability similar to the previous work [8, 13]. The amount was defined in two different ways: the number of steps and the AoM. The number of steps was measured by counting the heel strike events of each foot during the walking portion [14]. The AoM at each individual joint was calculated with the integration of absolute angular velocity at each joint over whole 1-h session including both gait and non-gait tasks as given by

$$ {AoM}_i={\int}_0^T\mid {\omega}_i(t)\mid dt $$

where ω_*i*_(*t*) is the angular velocity of *i* th joint, *T* is the final time of the session, and *i* represents each joint motion. The joint motions of interest included all three rotations of the pelvis and hip, as well as knee flexion/extension and ankle dorsi/plantarflexion, and were defined as individual AoMs. The total AoM was defined as the sum of all individual joint AoMs. The total AoM was additionally partitioned in gait (AoM_G_) and non-gait (AoM_NG_) periods and by unaffected (AoM_US_) and affected (AoM_AS_) sides. Intensity was estimated by the change in heart rate (*∆HR*) determined by average heart rate during therapy from the baseline during rest before the session [15]. Variability was defined as number of different tasks performed during the session recorded by the experimenter. Each session was composed of various tasks depending on the participant’s ability and therapist’s discretion.

We selected gait speed as the main functional outcome measure [8, 13]. In each session, we extracted a portion of therapy with normal, straight walking at a comfortable speed. The number of strides for each session was varied (between 5 to 80 strides) due to different impairment levels within and between subjects. The average gait speed was calculated by dividing average stride distance by the average time of gait cycles based on the lower body kinematic model with the anthropometric data estimated with height [16]. A simple correlation analysis showed a strong association between calculated gait speed and clinical outcome measure, the six-minute walk test (*r* = 0.91).

### Statistical analysis

R version 3.4.1 (2017 The R Foundation for Statistical Computing) was used for the statistical analysis. A linear mixed regression model was used to test the relationship between features of therapy dosage and average gait speed as a gait performance measure with significance level, *α* < 0.05. All features of dosage including AoM (total, partial or individual), step number, time, *∆HR*, number of different tasks performed were standardized and used as fixed effects and the subject was used as random effect of the mixed model. For the evaluation of better or best indicator of gait performance, we used comparison tools for mixed models including Akaike information criteria (AIC), Bayesian information criteria (BIC) and coefficient of determination (*R*^*2*^) [17]. To compare the difference in AoM between unaffected and affected sides, we used a paired t-test with significant level of *α* < 0.05.

## Results

### Overall dosage of therapy

**Fig. 2:**
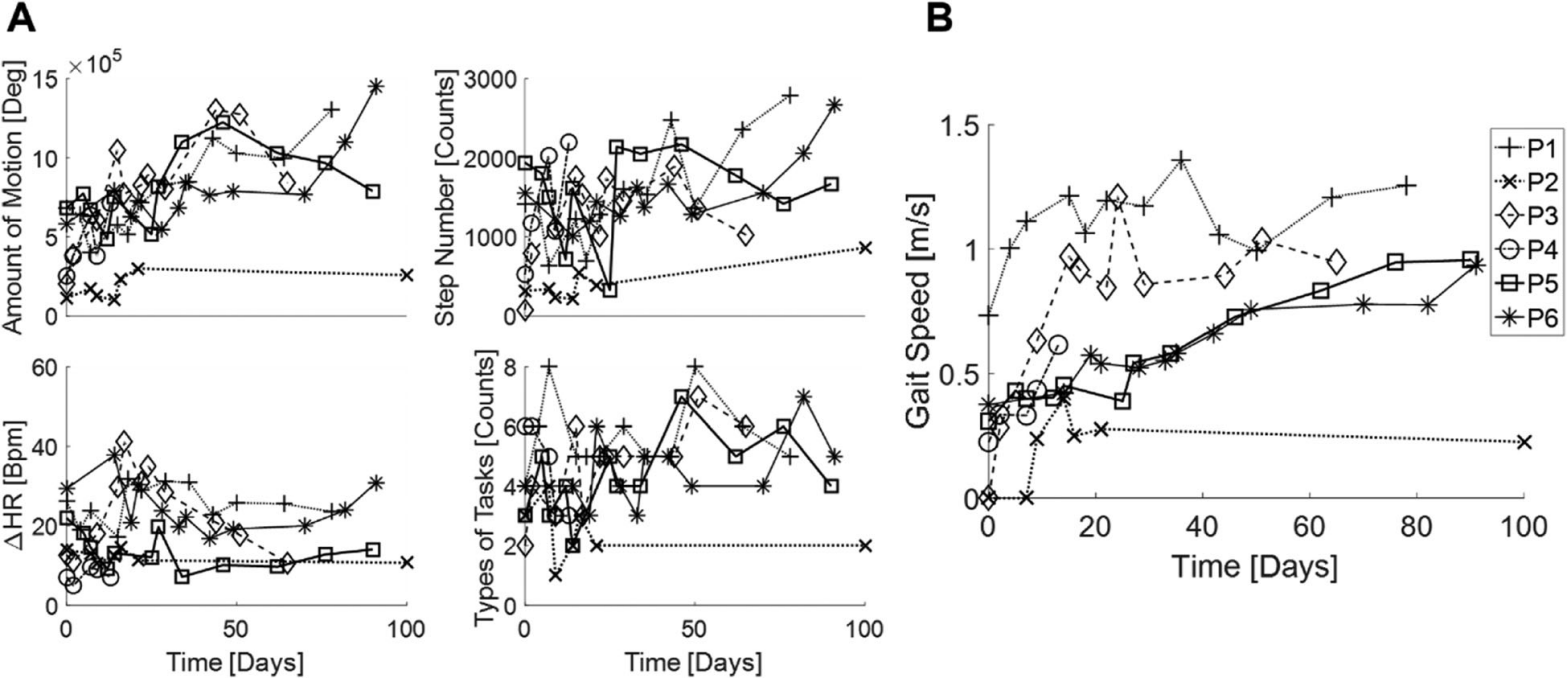
**a**, Changes in features of therapy dosage over time (top left) total amount of motion, (top right) step number, (bottom left) average change in heart rate from baseline, (bottom right) types of tasks performed for all subjects recorded. **b**, Changes in outcome measure of average gait speed over time

We first examined how the extracted features representing therapy dosage correlate with gait speed. Figure 2a and b illustrate changes over time in features of therapy dosage and the functional outcome, gait speed, respectively, for all six individuals. We used linear mixed regression models on these data for the statistical analysis. The results indicated all the independent variables were significantly correlated with gait speed, including total AoM (*p* < 0.01), step number (*p* < 0.01), types of tasks performed (*p* < 0.01), *∆HR* (*p* < 0.05), and time (*p* < 0.01). According to the goodness-of-fit measures, total AoM (*R*^*2*^ = 32.1%) revealed the greatest explained variance followed by time (*R*^*2*^ = 15.5%), step number (*R*^*2*^ = 14.1%), types of tasks performed (*R*^*2*^ = 8.0%) and *∆HR* (*R*^*2*^ = 5.8%) with consistent trends in AIC and BIC (smallest AIC and BIC for total AoM and greatest for *∆HR*). These results are summarized in Table 2.

**Table 2:** *p*-values and goodness-of-fit measures of linear mixed models with features of therapy dosage and average gait speed

	Fixed Effects	*β* [95% CI]	*R*^2^	AIC	BIC
Dosage of therapy features	*AoM*_*Total*_	0.534 [0.366, 0.703]***	32.1%^a^	105.9	114.3
	*Time*	0.413 [0.276, 0.549]***	15.5%	108.9	117.2
	*Steps*	0.374 [0.196, 0.552]***	14.1%^a^	121.6	129.9
	*Tasks*	0.275 [0.083, 0.468]**	8.0%	129.6	137.9
	*∆HR*	0.237 [0.003, 0.470]*	5.8%	133.4	141.8
AoM during gait period	*AoM*_*G*_	0.473 [0.305, 0.641]***	24.9%^a^	111.3	119.6
AoM during non-gait period	*AoM*_*NG*_	0.097 [−0.085, 0.279]	0.8%	136.4	144.7
AoM during gait and non-gait periods	*AoM*_*G*_ *AoM*_*NG*_	0.507 [0.343, 0.671]*** 0.173 [0.029, 0.318]*	32.1%^a^	107.5	117.9

*AoM*_*Total*_ total amount of motion, *∆HR* heart rate change, *AoM*_*G*_ amount of motion during gait, *AoM*_*NG*_ amount of motion during non-gait, *CI* confidence interval, *AIC/BIC* Akaike/Baysian Information Criterion with maximum likelihood, *** p <0.001, ** p < 0.01, * p < 0.05.

^a^Note that models with *AoM*_*Total*_ and *AoM* with both gait and non-gait portions (*AoM*_*G*_ and *AoM*_*NG*_) best represent the data as opposed to *AoM* during gait only (*AoM*_*G*_) and step number

### Amount of motion during gait and non-gait periods

We divided AoM into gait (AoM_G_) and non-gait (AoM_NG_) portions for each whole 1-h therapy session to analyze the differential effects of gait on recovery. On average, 45.5% of the duration of the therapy session was dedicated to gait training, and 75.3% of the total AoM during a therapy session occurred during walking. Table 2 shows the results of models with AoM_G_ and AoM_NG_. AoM_G_ demonstrated a significant association with gait speed (*p* < 0.01) whereas AoM_NG_ alone did not (*p* =0.29). However, the model including both AoM_G_ and AoM_NG_ showed both parameters significantly correlated with gait speed (*p* < 0.01 for AoM_G_ and *p* < 0.05 for AoM_NG_). Further, the variance accounted for the latter model (*R*^*2*^ = 32.1%) was higher than AoM_G_ alone (*R*^*2*^ = 24.9%) and AoM_NG_ alone (*R*^*2*^ = 0.8%). We also compared AoM_G_ alone with step number. While they both revealed significant associations with gait speed (both *p* < 0.01), the variance explained by AoM_G_ (*R*^*2*^ = 24.9%) was greater than step number (*R*^*2*^ = 14.1%). Additional correlation analyses demonstrated stronger association between AoM_G_ and step number (*r* = 0.93) than total AoM with step number (*r* = 0.83).

### Amount of motion at individual joints

We additionally observed how specific joint motions were correlated with functional gait recovery. Because joint AoMs were not independent (average *r* = 0.86), we modeled each joint motion alone instead of multiple joint AoMs. Results are presented in Table 3. All individual joints were significantly correlated with the average gait speed (*p* < 0.01). Goodness-of-fit measures varied between *R*^*2*^ = 16.5–31.2%, the greatest at hip abduction/adduction and smallest at hip internal/external rotation, both of the unaffected sides.

**Table 3:** *p*-values and goodness-of-fit measures of linear mixed models with partial and individual AoMs and average gait speed

	Fixed Effects	*β* [95% CI]	*R*^2^	AIC	BIC
AoM of US/AS	*AoM*_*US*_	0.536 [0.364, 0.708]***	32.9%^a^	106.9	115.2
	*AoM*_*AS*_	0.503 [0.337, 0.668]***	27.8%^a^	107.9	116.2
AoM of individual joints at pelvis	*AoM*_*Pel*, *tilt*_	0.531 [0.343, 0.718]***	30.6%^b^	111.1	119.4
	*AoM*_*Pel*, *oblq*_	0.500 [0.319, 0.681] ***	27.4%	112.1	120.4
	*AoM*_*Pel*, *ro*_	0.429 [0.234, 0.623] ***	19.0%	120.2	128.5
AoM of individual joints at US	*AoM*_*hip*, *abd*_	0.547 [0.371, 0.723] ***	31.2%^b^	106.9	115.2
	*AoM*_*hip*, *ie*_	0.383 [0.184, 0.583] ***	16.5%	124.0	132.3
	*AoM*_*hip*, *fe*_	0.457 [0.266, 0.647] ***	24.8%	117.8	126.2
	*AoM*_*knee*, *fe*_	0.509 [0.353, 0.665] ***	29.2%^b^	104.5	112.8
	*AoM*_*ankle*, *pdf*_	0.470 [0.301, 0.639] ***	24.1%	111.9	120.2
AoM of individual joints at AS	*AoM*_*hip*, *abd*_	0.427 [0.252, 0.603] ***	19.8%	117.0	125.3
	*AoM*_*hip*, *ie*_	0.403 [0.223, 0.582] ***	16.7%	119.6	127.9
	*AoM*_*hip*, *fe*_	0.451 [0.276, 0.626] ***	23.5%	114.9	123.2
	*AoM*_*knee*, *fe*_	0.520 [0.364, 0.675] ***	28.1%^b^	103.4	111.7
	*AoM*_*ankle*, *pdf*_	0.484 [0.320, 0.648] ***	26.0%	109.3	117.6

*US* unaffected side, *AS* affected side, *Pel* pelvis, *oblq* obliquity, *ro* rotation, *abd* abd/adduction, *ie* int/external rotation, *fe* flex/extension, *pdf* plantar/dorsiflexion, *** < 0.001

^a^Note better representation of data on unaffected side compared to affected side

^b^Also, joints with various ranges of motion (knee, hip abduction, pelvic tilt) represent data best on joint level (see Discussion section)

### Amount of motion between unaffected and affected sides

Next we determined the degree to which the motion on the affected and unaffected sides corresponded to recovery. Both AoM_US_ and AoM_AS_ were correlated with functional gait recovery (both *p* < 0.01, see Table 3). However, the goodness-of-fit measures demonstrated slightly greater explained variance in AoM_US_ (*R*^*2*^ = 32.9%) compared to AoM_AS_ (*R*^*2*^ = 27.8%) with similar trends in AIC and BIC. Secondly, paired t-tests were used to evaluate absolute differences between AoM_US_ and AoM_AS_ as well as individual joints (i.e., hip flexion/extension, abduction/adduction, internal/external rotation, knee flexion/extension and ankle plantar/dorsi-flexion) of bilateral legs (see Table 4). There were no significant differences between AoM_US_ and AoM_AS_, and individual joints (all *p* > 0.05) except for ankle plantar/dorsiflexion with greater AoM at unaffected side (*p* < 0.05).

**Table 4:** AoM of the differences between unaffected and affected sides over all sessions. Differences evaluated with paired t-test

	*∆AoM* [95% CI]	*p*-value
*AoM*_*US*/*AS*_	22,911 ° [− 4659, 50,481]	0.09
*AoM*_*hip*, *abd*_	− 867 ° [− 3154, 1420]	0.37
*AoM*_*hip*, *ro*_	− 5112 ° [− 14,125, 3901]	0.20
*AoM*_*hip*, *fe*_	5536 ° [− 3582, 14,654]	0.18
*AoM*_*knee*, *fe*_	10,870 ° [− 1199, 22,939]	0.07
*AoM*_*ankle*, *pdf*_	12,484 ° [2592, 22,376]	< 0.05

*∆AoM* = *AoM*_*US*_ − *AoM*_*AS*_

## Discussion

The “dosage” that physical therapy provides is one of the most fundamental but least understood phenomena in rehabilitation. In this study, we take the novel approach quantifying therapy using portable lower limb motion capture. Additionally, we longitudinally recorded therapy sessions during both inpatient and outpatient phases in order to measure as early and extensively as possible. Our main finding was that the amount of motion (AoM) revealed greater association with gait speed than the number of steps, spontaneous recovery (i.e. time), intensity parameterized with heart rate change or the types of tasks performed. This result and the additional findings outlined in this study suggest that wearable sensors can become a valuable tool for better understanding dosage of therapy received and comparing the efficacy of different rehabilitation training regimes.

Several early studies attempted to quantify dosage of therapy by observing duration or number of repetitions in task-specific movements [4, 5, 18, 19]. More recent studies measured overall limb motions using accelerometers and concluded that the number of steps was associated with improved gait recovery [7, 8, 13]. However, we hypothesized that richer data involving joint motions would reveal greater nuance and thus result in a more accurate correlate of recovery. We observed that the AoM was substantially more associated with recovery (*R*^*2*^ = 32.1%) than number of steps (*R*^*2*^ = 14.1%). This result suggests that obtaining joint kinematics during therapy results in a useful indicator of recovery. Since this was an observational study, we cannot conclude whether inducing greater AoM will result in improved recovery. Yet we speculate that this relationship would likely be found given the greater walking outcomes in the high intensity therapy group compared to the control group who received conventional therapy [8].

It is possible that the high correlation of AoM with gait speed is due to the simple observation that walking faster results in greater joint motion. Our results suggest that this is not the case. We separated the AoM during gait (AoM_G_) from AoM during activities other than gait (AoM_NG_, e.g. transfers, stretching/balancing, exercise bike, etc.). If gait speed was primarily a reflection of the amount of motion during gait of a therapy session, we would expect the highest correlation between AoM_G_ and gait speed. On the contrary, the model (Table 2) that accounted for the greatest variance in gait speed included both AoM_G_ and AoM_NG_, suggesting that the motions during non-gait tasks were important in explaining recovery. This corresponds with previous finding that the exercise dose including both gait and non-gait training in the early stage is an important indicator of walking speed [20]. Further, while AoM_G_ associated strongly with step number (*r* = 0.93), total AoM was not as strong (*r* = 0.83) despite being a better correlate of gait recovery than step number. Thus, these results suggest that AoM may provide additional valuable information not included in step number.

In addition to AoM, other features including time, step number, change in heart rate, and types of tasks were also significantly correlated with gait speed, consistent with previous work [8, 13, 21]. The correlations of these parameters indicate that they may also be useful features that represent functional recovery. However, we surprisingly observed that time (*R*^*2*^ = 15.5%), representative of spontaneous recovery, explained more variance than the other variables including step number (*R*^*2*^ = 14.1%). Further research is needed on a larger sample to obtain a more holistic perspective on these relations.

While recording kinematics of full lower body segments provides a rich kinematic dataset, it is possible we could get similar explained value from fewer sensors given that all individual joint AoMs significantly associated with gait speed (all *p* < 0.001, see Table 3). We found the greatest explained variance in unaffected side hip abduction/adduction (*R*^*2*^ = 31.2%) followed by pelvic tilt (*R*^*2*^ = 30.6%), and bilateral knee flexion/extension of the unaffected (*R*^*2*^ = 29.2%) and affected (*R*^*2*^ = 28.1%) sides. It is unclear why hip abduction/adduction of the unaffected side appeared to be so influential given that it is not typically a focus of treatment or indicative of locomotor function. This result may be related to the strong correlation between range of motion at unaffected side hip abduction/adduction and gait speed in stroke individuals [22]. However, further research is needed to investigate this relationship, which may indeed be epiphenomenal. On the other hand, pelvic tilt is highly correlated to gait speed, and its increased range of motion would indicate greater fore/aft balance [23]. The inclusion of knee flexion/extension is expected as this joint has long been believed a key contributor of functional activities including walking [24]. These results suggest that a single IMU on the pelvis, an additional one on the unaffected thigh, or measuring knee joint motions could provide similar explained variance for this application as a full lower body suit.

We observed that AoM of the unaffected side explained more variance than the affected side (Table 3). This finding was in contrast to our expectation that the affected side would have a greater association with functional recovery. Despite no clear difference in the AoM between sides (except for ankle dorsi/plantarflexion likely explained by restricted motion from AFO, see Table 4), the unaffected side accounted for 32.9% of the variance compared to 27.8% of the affected side. This result may be supported by previous studies that found greater correlations between gait speed and selected joint variables (i.e., joint range, moment, power, muscle strength) of the unaffected lower limb than affected side with chronic post-stroke individuals [22, 25]. While this result may simply be due to compensatory action of the unaffected limb, it is feasible that physiological coupling between the limbs may play a role in this effect as well [26]. While delineation of the role of the unaffected limb in recovery requires further investigation, the result implies the richer information able to be obtained using portable motion capture compared to accelerometers on the foot.

While here we have focused on functional recovery (i.e., gait speed), true recovery is the restoration of the manner in which the movement was originally made [27]. This definition does not necessarily match with improved gait speed or what is often considered “recovery” from a clinical perspective, i.e. the ability to achieve an activity of daily living. For example, increased pelvic obliquity or hip circumduction compensates for lack of foot clearance [28]. These motions may prevent tripping and speed walking, suggesting that the patient has recovered, but the energy cost increases exponentially with circumduction amplitude [29]. Thus, while such compensations facilitate some base level of function, the level of function still remains below healthy human ability. Monitoring patients over the course of early recovery can quantitatively detail phenomena such as this, as well as important concepts such as differential effects of paretic vs. non-paretic limb compensations [30] that are currently unaccounted for. This information can be used to refine a prescribed treatment and possibly help update the clinical classification of recovery.

The purpose of this proof-of-concept study was to provide initial evidence that recording kinematics during therapy can provide more valuable information than previously measured quantities such as step number. Our observations suggest that it can. However, our conclusions are limited to a small sample size only including individuals who were capable of walking within the initial stage of recovery. The average length of inpatient hospital stay in US is approximately 3 weeks [31], and many of the patients were not able to continue the outpatient therapy at same institute after discharge (e.g., location, insurance coverage, move to other facilities or receiving home therapy, etc.). Despite the small sample size, our data still provided clear initial results on the relative importance of several indicators, offering a glimpse into how therapy can be effectively monitored in the future. However, expanding this work incorporating a larger range of impairment levels, possibly through a network of hospitals, would overcome these hurdles and improve generalizations.

Our conclusions are also limited to the assumption that gait speed is the primary measure of gait recovery [8]. We did not include clinical measures such as Functional Independence Measure (FIM), Berg balance scale or 5X sit-to-stand [32] here because these measures were not acquired every therapy session and thus were not helpful in explaining variance. However, future work could incorporate these clinical outcomes to help better describe gait recovery.

There were also technical limitations that may have affected our results. IMUs have historically shown inaccuracies compared to optical motion capture [33]. We tried to mitigate these issues by using an IMU system specifically designed for capturing kinematics and has been validated [10, 11, 34]. Also, relatively short battery life of our system compared to simple accelerometers restricted to measure the activities beyond therapy sessions. However, the activities in daily life beyond therapy may have affected recovery especially during outpatient phase. We expect that future advances in gait-centered IMU technology including prolonged battery life will lead to improved understanding of therapy dosage with more accurate and broader data collection.

## Conclusions

Despite decades of research on physical therapy, we still lack knowledge of is primary functional elements. In this work we introduced a novel approach of unobtrusively recording lower body kinematics longitudinally during post-stroke therapy. We conclude that the amount of joint motion during therapy can be an important indicator of recovery (gait speed) in post-stroke individuals. Portable motion capture using IMUs can provide valuable data and greater insight into the therapy experience. These promising initial results justify further research into the dosage of therapy in a larger clinical study.

## Data Availability

Data supporting the conclusions of this manuscript is included within the manuscript. The data collected in this study are available from the corresponding author on reasonable request.

## Abbreviations

*∆HR*: Change in heart rate
AFO: Ankle foot orthosis
AIC: Akaike information criteria
AoM: Amount of motion
BIC: Bayesian information criteria
FIM: Functional independence measure
IMU: Inertial measurement unit
